# Spatiotemporal analysis of HPV vaccination and associated neighborhood-level disparities in Texas—an ecological study

**DOI:** 10.3389/fpubh.2024.1418526

**Published:** 2024-06-25

**Authors:** Ryan Ramphul, Abigail S. Zamorano, Saswati Upadhyay, Manali Desai, Cici Bauer

**Affiliations:** ^1^Department of Epidemiology, The University of Texas Health Science Center at Houston School of Public Health, Houston, TX, United States; ^2^The Joint Collaborative on Geospatial Analysis and Health, A Collaboration of The University of Texas Health Science Center at Houston School of Public Health and The University of Texas MD Anderson Cancer Center, Houston, TX, United States; ^3^Division of Gynecologic Oncology, Department of Obstetrics, Gynecology, and Reproductive Sciences, The University of Texas Health Sciences Center at Houston, McGovern Medical School, Houston, TX, United States; ^4^Department of Environmental and Occupational Sciences, The University of Texas Health Science Center at Houston School of Public Health, Houston, TX, United States; ^5^Department of Biostatistics and Data Science, The University of Texas Health Science Center at Houston School of Public Health, Houston, TX, United States; ^6^Center for Spatial-Temporal Modeling for Applications in Population Sciences,University of Texas Health Science Center at Houston School of Public Health, Houston, TX, United States

**Keywords:** spatial analysis, HPV vaccination, vaccination disparities, cancer prevention, minority health, HPV, geospatial analysis, health care disparities

## Abstract

**Background:**

HPV is responsible for most cervical, oropharyngeal, anal, vaginal, and vulvar cancers. The HPV vaccine has decreased cervical cancer incidence, but only 49% of Texas adolescents have initiated the vaccine. Texas shows great variation in HPV vaccination rates. We used geospatial analysis to identify areas with high and low vaccination rates and explored differences in neighborhood characteristics.

**Methods:**

Using Anselin’s Local Moran’s I statistic, we conducted an ecological analysis of hot and cold spots of adolescent HPV vaccination coverage in Texas from 2017 to 2021. Next, we utilized a Mann–Whitney U test to compare neighborhood characteristics of vaccination coverage in hot spots versus cold spots, leveraging data from the Child Opportunity Index (COI) and American Community Survey.

**Results:**

In Texas, there are 64 persistent vaccination coverage hotspots and 55 persistent vaccination coverage cold spots. The persistent vaccination coverage hot spots are characterized by ZIP codes with lower COI scores, higher percentages of Hispanic residents, higher poverty rates, and smaller populations per square mile compared to vaccine coverage cold spots. We found a more pronounced spatial clustering pattern for male adolescent vaccine coverage than we did for female adolescent vaccine coverage.

**Conclusion:**

In Texas, HPV vaccination coverage rates differ depending on the community’s income level, with lower-income areas achieving higher success rates. Notably, there are also gender-based discrepancies in vaccination coverage rates, particularly among male adolescents. This knowledge can aid advocates in customizing their outreach initiatives to address these disparities.

## Introduction

Human papillomavirus (HPV) accounts for over 99% of cervical cancers and most oropharyngeal, anal, vaginal, and vulvar cancer (1, 2). More than 90% of HPV-associated cancers are preventable through HPV vaccination (3), which is recommended as a routine vaccination for children as early as age 9, with catch-up recommended up to age 26 for those not previously vaccinated and up to age 45 using shared clinical decision-making (4). Since the introduction of the HPV vaccine in 2006, there has already been a significant decline in nationwide cervical cancer incidence, especially in the 15–20-year age group, suggesting the positive impact of vaccination (5). However, the United States falls well behind its Healthy People 2020 target of 80% vaccinated adolescents, with only 54.5% completing the series (6, 7).

As of 2017, only 49% of adolescents in Texas had initiated the HPV vaccine series, and the state ranks 47 out of 50 in vaccination rates nationwide (8). Vaccination rates across the state are not geographically uniform, with some rural counties outperforming the major cities and the rest of the states (8, 9). While previous studies have demonstrated this regional variation, they have been limited to analyses of large state regions, which makes further analysis of associated sociodemographic factors incomplete.

Geospatial analyses of HPV vaccination have been crucial to identify disparities and target areas for intervention. A systematic review of area-level variation in HPV vaccination uptake revealed significant differences influenced by socioeconomic factors, healthcare access, and educational attainment (10). For example, regions with higher poverty rates and lower access to healthcare services often exhibit lower vaccination rates. Conversely, areas with robust public health infrastructures and targeted education campaigns tend to achieve higher vaccination coverage. These findings emphasize the need for localized public health strategies considering these sociodemographic factors. By understanding the specific characteristics and barriers in different regions, public health initiatives can be better tailored to improve HPV vaccination rates effectively (10, 11). Such detailed geospatial and sociodemographic analyses can help bridge the gaps in HPV vaccination coverage, ensuring more uniform protection against HPV-associated cancers across diverse communities.

Significant barriers to HPV vaccination exist, including lack of knowledge of the vaccine or the associated cancers, lack of access to immunization (involving geographic, financial, and public policy factors), lack of provider recommendation, and parental hesitations about accepting the vaccine for children/adolescents due to the association with a sexually transmitted infection (12). We aim to geospatially model areas of Texas with persistently high or low levels of HPV vaccination coverage to better understand the associated area-level characteristics and identify areas where increased vaccination efforts may be pursued.

## Methods

### Study population

Data were obtained through the Texas Department of State Health Services Immunization Information System (ImmTrac2) Registry (13) on the percentage of registrants aged 9+ who received at least one dose of the HPV vaccine, categorized by ZIP code, for each year from 2017 to 2021. It also provided separate percentage estimates of male and female registrants aged 9+ who received at least one dose by ZIP for each year from 2017–2021. ImmTrac2 is a state-wide opt-in vaccine registry and includes information provided by healthcare providers, pharmacies, public health clinics, Medicaid claims administrators, and the Texas Department of State Health Services Vital Statistics Unit. While it contains information for both children and adults, most children are entered into the system at birth, while adults over 18 must consent to participate or continue participation. Additionally, vaccinations given to children must be reported by law (13). As a result, the ImmTrac2 registry is predominantly valuable as a database for vaccinations of childhood and adolescence. ImmTrac2 opt-in immunization registry requires parental consent to store children’s vaccination records. While Texas law mandates healthcare providers to report all immunizations given to children under 18 to ImmTrac2, including these records in the registry relies on obtaining parental or guardian consent. If consent is provided, the vaccination information is stored; otherwise, it is not, although the provider still meets the legal reporting requirement. The exact percentage of childhood vaccinations reported to ImmTrac2 varies based on consent rates, but mandatory reporting ensures a high overall reporting rate despite the lack of precise figures (13). In 2020, 80% of Texas children under 6 years old had at least two immunizations recorded in ImmTrac2 (14), indicating ImmTrac2’s robust coverage of pediatric populations.

Data in this study was publicly available and received an exemption from Institutional Review Board (IRB) approval.

### Mapping and statistical analysis

We utilized Anselin’s Local Moran’s I statistic, pioneered by Luc Anselin in the “Local Indicators of Spatial Association—LISA,” to identify statistically significant clusters of ZIP codes with high/low estimated rates of HPV vaccination coverage each year from 2017 to 2021 (15). Using the Environmental Science Research Institute’s ArcGIS Pro Version 2.2.0 software, we ran the Cluster and Outlier Analysis Tool, which implements the Local Moran’s I statistic by first determining a “neighborhood” around each ZIP code in the dataset (16). While several strategies exist to determine the “neighborhood” around each ZIP code, we used the Queen Contiguity Method. In this method, all ZIP codes that touch a ZIP code are considered its “neighborhood” and are included in its computations (17). The Queen Contiguity Method has been successfully used in several studies to identify health outcomes and service clusters (17).

After determining the neighborhoods around each ZIP code, the Cluster and Outlier Analysis Tool calculates a Local Moran’s I score for each ZIP code, where a positive value for “I” indicates that a ZIP code has a neighboring ZIP code with similarly high or low vaccination rates compared to the rest of the study area (16). These ZIP codes are part of clusters. After calculating a Local Moran’s I for each ZIP code in the data set, statistical significance is tested by running a Monte Carlo simulation. The values in the “neighborhoods” around each ZIP in the study area are randomly rearranged 9,999 times. A Local Moran’s I score is calculated each time, creating a random reference distribution of Local Moran’s I to compare with the observed Local Moran’s I. A pseudo-*p*-value is then calculated by determining the proportion of Local Moran’s I statistics generated from random permutations that display more clustering than the original data. If the proportion is less than 0.05, the ZIP code is demarcated as a statistically significant vaccination coverage hot or cold spot (16). Persistent vaccination coverage in hot and cold spots were defined as ZIP codes that consistently exhibited statistically significant high or low vaccination coverage rates over five consecutive years, respectively.

Finally, we utilized a Mann–Whitney U test to explore statistical differences in neighborhood characteristics between ZIP codes that were persistent vaccination coverage hot or cold spots for all registrants aged 9+ and then by male and female registrants. Median percentages of neighborhood characteristics (e.g., % Black, % below the federal poverty level, etc.) were chosen as the measure of the center instead of the mean because it is less affected by extreme values and skewed distributions. This provides a more accurate representation of the central tendency in our dataset, where variables like income and population density exhibit significant skewness. Statistical analyses were performed in SPSS version 28.01.1.

### Neighborhood evaluation metrics

The Child Opportunity Index (COI) and the U.S. Census Bureau’s American Community Survey (ACS) were used to describe neighborhood characteristics (18, 19). The COI is a validated composite index to measure neighborhood resources and conditions. It consists of 29 social determinants of health (SDOH) indicators of neighborhood-based opportunities, including high-quality schools, green space, healthy food, toxin-free environments, and socioeconomic resources (18). COI indicators are assigned individual z-scores and summed to an overall z-score using indicator-specific weights that signify how strongly each indicator predicts children’s health and economic outcomes. We used the most recent version of this data, which was from 2015. The COI indicators are divided into three subdomains: Education, Health and Environment, and Social and Economic. ZIP codes are scored as a very low opportunity, low opportunity, moderate opportunity, high opportunity, or very high opportunity within each domain (18).

The ACS is the U.S. Census Bureau’s largest household survey and provides ZIP code level estimates of poverty levels, racial/ethnic diversity, insurance coverage, and population density (19). We used the most recently published data (2016–2020), which gives an average estimate of the characteristics over the 60-month period. Finally, we condensed ZIP code level Rural–Urban Commuting Area (RUCA) codes (20) into four categories (urban-focused, large rural city, small rural town, and isolated small rural town) as discussed by the Rural Health Research Center.

## Results

**Figure 1:**
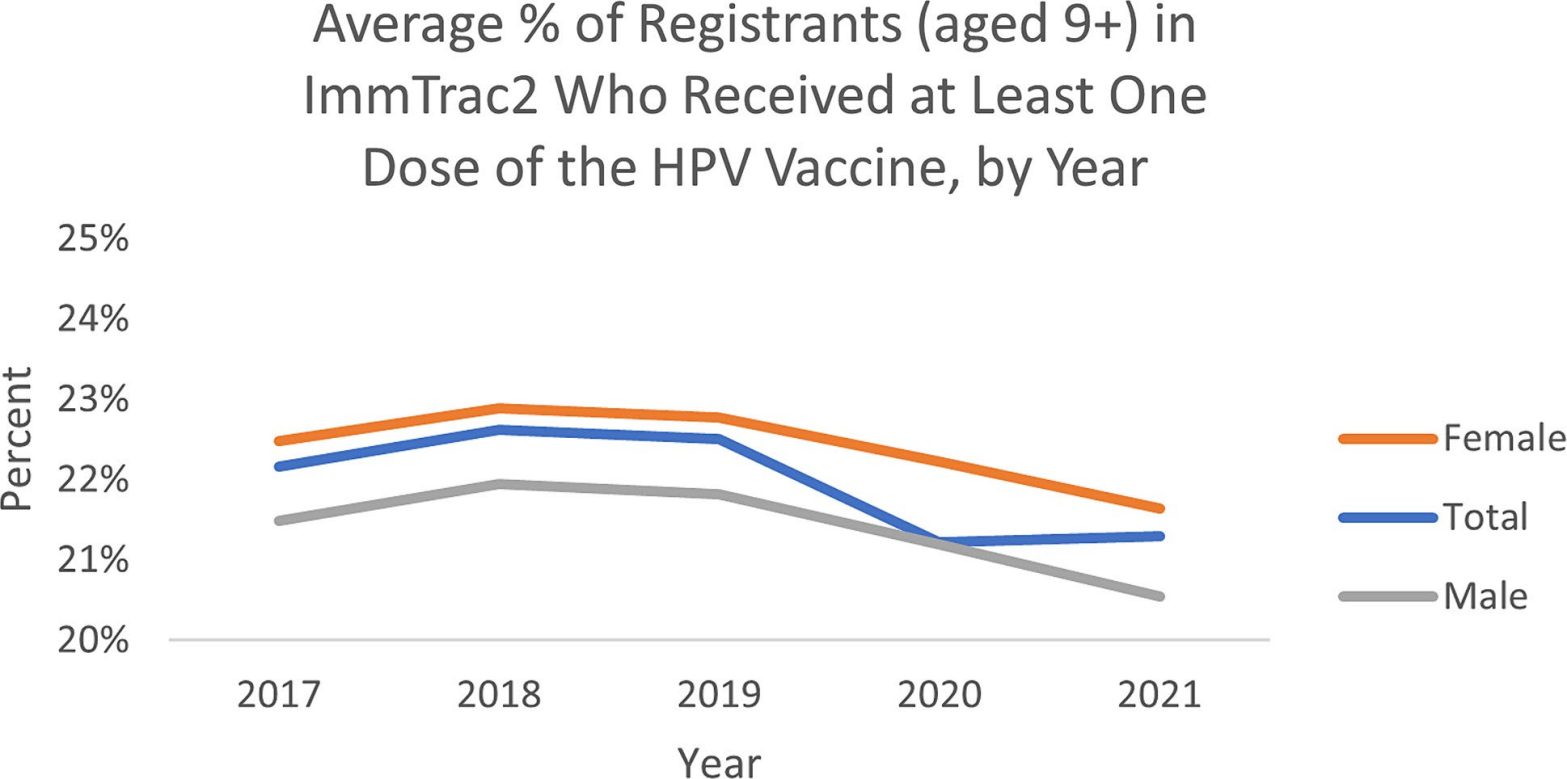
Average percent of registrants (aged 9+) by ZIP in ImmTrac2, who received at least one dose of the HPV vaccine in Texas.

The average percent of registrants (aged 9+) by ZIP code in Texas’s ImmTrac2 database who received at least one dose of the HPV vaccine remained relatively similar from 2017 to 2021, though trended slightly downward over the 5 years (Figure 1). The average percentage ranged between 21 and 23%. Average vaccination rates by ZIP over this five-year period were higher for female adolescents than male adolescents, which aligns with trend data from the CDC’s National Immunization Surveys (11).

**Figure 2:**
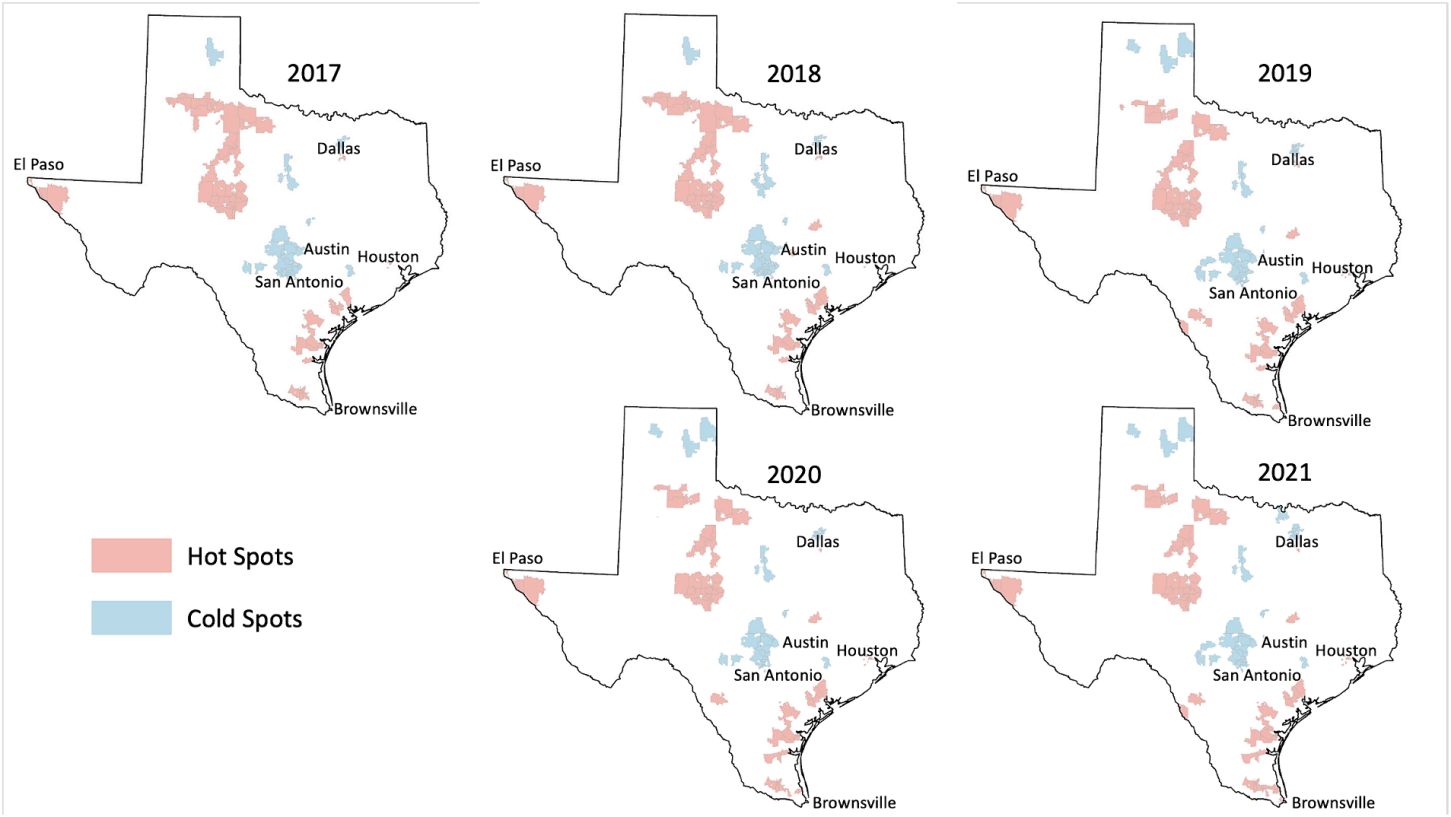
HPV vaccination hot and cold spots in Texas.

**Figure 3:**
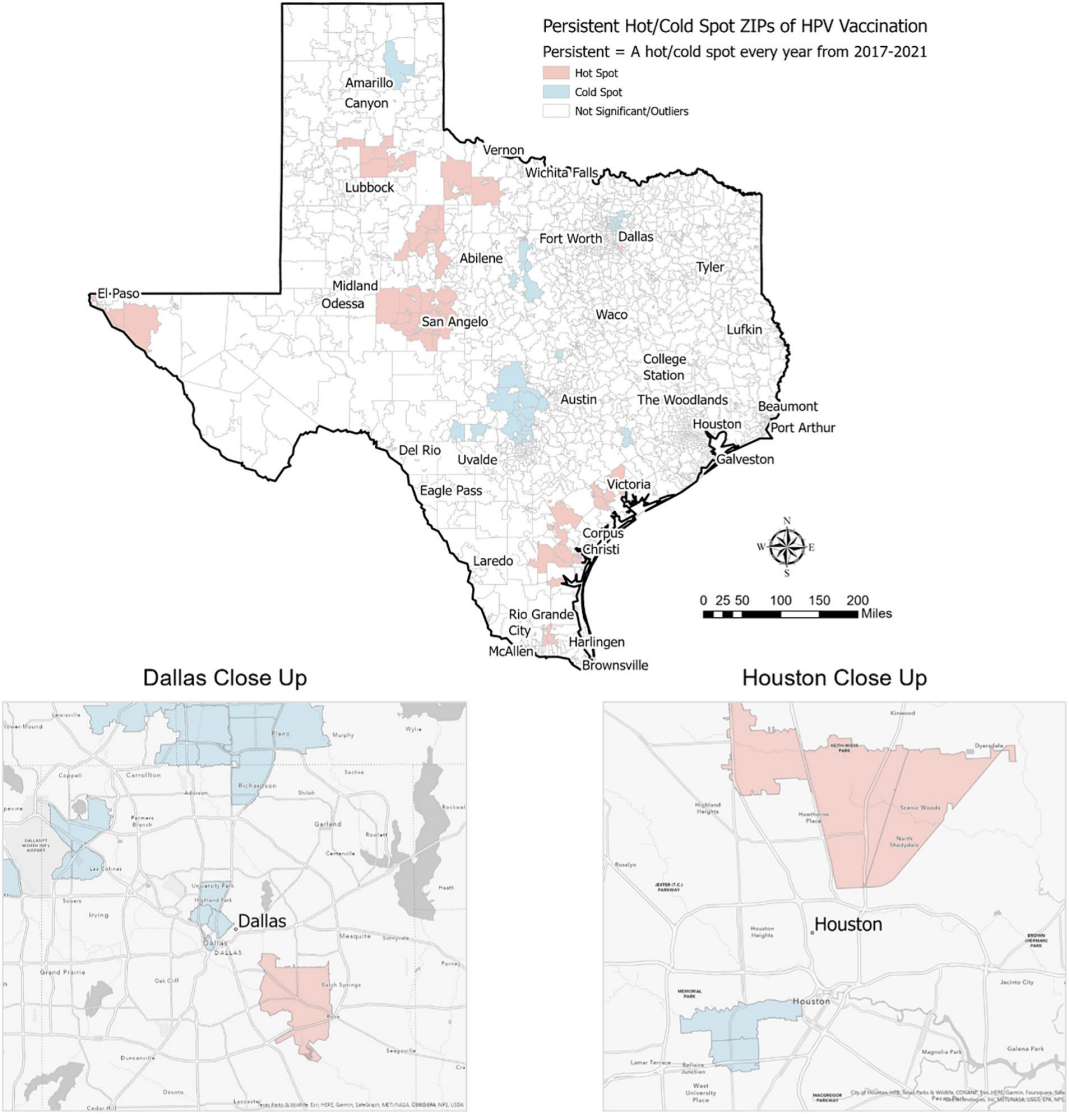
Persistent hot/cold spot ZIP codes for HPV vaccination in Texas, 2017–2021.

Among the 1,800+ ZIP codes in Texas, we identified 64 ZIP codes that were statistically significant hot spots of HPV vaccination coverage 5 years in a row and 55 ZIP codes that were cold spots of vaccination coverage 5 years in a row. As illustrated in Figures 2, 3, the persistent vaccination coverage hot spots were primarily near the southern Gulf coast, the northwestern portion of the state, and close to El Paso. Cold spots were primarily located in the central portion of the state, the panhandle, and in parts of major urban areas like Houston and Dallas. However, on closer inspection of the major cities of Dallas and Houston, there are significant differences in these densely populated areas, with the cold spots in the urban and suburban wealthier areas and the hot spots in southeast Dallas and northeast Houston, which are typically lower income. The remainder of the ZIP codes in the state were not part of statistically significant clusters 5 years in a row or were outliers.

When evaluating differences in HPV vaccination by reported gender, we found 55 hot spot ZIP codes and 38 cold spot ZIP codes among male adolescents alone. These were in areas similar to overall hot/cold spots. However, among female adolescents alone, we identified just 11 hot spot ZIP codes of HPV vaccination and 13 cold spot ZIP codes. These were also located in areas like overall hot/cold spots (figures by gender not shown).

### Demographic analysis of persistent hot spot and cold spot ZIPs

We found that persistent hot spot ZIP codes of HPV vaccination coverage had significantly lower overall COI scores, indicating lower opportunity areas than persistent cold spot ZIP codes (overall median score 2 vs. 4, *p* < 0.001, Table 1). As seen in Figure 2, Temporal changes in both vaccination hot spots and cold spots were identified. Over time, hot spots on the bottom part of the southern Gulf Coast expanded, whereas the northwestern portion has decreased. Cold spots, especially in the panhandle, expanded over time. Overall, spatial patterns were quite similar. This difference persisted in each subdomain (Education, Health, Environment, and Social and Economic) and when each was broken out by gender (Table 1).

**Table 1:** Child opportunity index score analysis of persistent hot and cold spot ZIPs of HPV vaccination.

Child opportunity index domains^†^	Persistent hot spots	Persistent cold spots	*p*-value*
Child opportunity index score, overall (median score)	2.0	4.0	**<0.001**
Males aged 9+	2.0	4.0	**<0.001**
Females aged 9+	3.0	5.0	**0.003**
Education domain (median score)	2.0	5.0	**<0.001**
Males aged 9+	2.0	3.5	**<0.001**
Females aged 9+	3.0	5.0	**0.009**
Health and environment domain (median score)	2.0	4.0	**<0.001**
Males aged 9+	2.0	4.0	**<0.001**
Females aged 9+	3.0	5.0	**<0.001**
Social and economic domain (median score)	2.0	4.0	**<0.001**
Males aged 9+	2.0	4.0	**<0.001**
Females aged 9+	3.0	5.0	**0.026**

^†^1, very low opportunity; 2, low opportunity; 3, moderate opportunity; 5, high opportunity; 6, very high opportunity. *Mann–Whitney U Test Significance Level (0.05). Bold text indicate statistically significant values.

As shown in Table 2, using American Community Survey (ACS) data, persistent hot spot ZIPs had a higher median percentage of the population below the federal poverty level (17.40% vs. 7.14%, *p* < 0.001). Persistent hot spots also had higher rates of resident children who are either uninsured (2.87% vs. 1.92%, *p* < 0.001) or on public insurance (11.75% vs. 2.91%, *p* < 0.001) than persistent cold spots. Cold spot ZIPs had statistically higher percentages of children on private insurance plans (12.3% vs. 8.6%) (*p* = 0.001). There were significantly higher rates of Hispanic residents in hot spot ZIPs compared to cold spot ZIPs (53.32% vs. 20.65%, *p* < 0.001) and lower percentages of Asian residents (0.89% vs. 8.28%, *p* < 0.001). Persistent hot spot ZIPs did have a statistically higher rate of Caucasian residents than persistent cold spots, but this did not persist when broken out by gender. There was no difference in the percentage of Black residents overall or by gender between hot and cold spots. Hot spot ZIPs had lower median populations per square mile (29.00 vs. 2287.80, *p* < 0.001). Still, there was no difference in rural/urban designation by RUCA code, with the majority being urban-focused.

**Table 2:** Demographic and socioeconomic characteristics of persistent hot spots and cold spots.

ZIP code level characteristics		Persistent hot spots	Persistent cold spots	*p*-value*
Income	Population below the federal poverty level (percentage, median)	17.4	7.1	**<0.001**
	Males aged 9+	17.6	9.5	**<0.001**
	Females aged 9+	12.2	8.4	**0.007**
	Median household income (USD, median)	47,751	83,486.0	**<0.001**
	Males aged 9+	46,880	71,999.5	**<0.001**
	Females aged 9+	58,795	85,942	**<0.001**
Race/Ethnicity (percentage, median)	Caucasian	87.8	73.3	**0.025**
	Males aged 9+	84.98	79.8	0.566
	Females aged 9+	88.3	73.3	0.459
	African American	3.2	7.9	0.223
	Males aged 9+	4.6	5.1	0.842
	Females aged 9+	4.3	7.9	0.649
	Hispanic	53.3	20.7	**<0.001**
	Males aged 9+	67.4	20.96	**<0.001**
	Females aged 9+	38.98	22.3	**0.041**
	Asian	0.89	8.3	**<0.001**
	Males aged 9+	0.9	4.1	**0.007**
	Females aged 9+	0.7	8.6	**0.035**
Insurance coverage of children (percentage, median)	Medicaid	11.8	2.9	**<0.001**
	Males aged 9+	13.3	3.5	**<0.001**
	Females aged 9+	8.2	2.8	**<0.001**
	Private	8.2	12.3	**0.001**
	Males aged 9+	7.9	9.9	**0.029**
	Females aged 9+	11.1	13.5	0.277
	Uninsured	2.9	1.9	**<0.001**
	Males aged 9+	3.1	1.9	**0.009**
	Females aged 9+	3.02	1.9	0.150
Population density	Population per square mile (median)	29.0	2287.80	**<0.001**
	Males aged 9+	41.5	168.9	**0.012**
	Females aged 9+	5.5	199.04	**0.004**
	RUCA codes (median result)^‡^	Urban-focused	Urban-focused	0.445
	Males aged 9+	Urban-focused	Urban-focused	0.124
	Females aged 9+	Urban-focused	Urban-focused	0.649

*Mann–Whitney U Test Significance Level (0.05). ^‡^Categorized into four domains: Urban-focused, Large rural city, Small rural town, and Isolated rural. Bold text indicate statistically significant values.

## Discussion

This study found significant geographic, gender, and socioeconomic disparities between persistent hot spots and persistent cold spots of HPV vaccination coverage in Texas. Persistent hot spots are more likely to be disadvantaged neighborhoods, with lower Child Opportunity Index scores and subdomain scores, higher poverty rates, lower median household incomes, and greater percentages of children on public insurance or uninsured. They are also generally less densely populated than persistent cold spots and have higher percentages of Hispanic residents. Persistent cold spots of HPV vaccination, on the other hand, have significantly higher rates of Asian residents and are more densely populated. This finding aligns with studies documenting a “reverse disparity” in HPV vaccination, with higher rates among certain racial minorities and those receiving public insurance (21). It has been hypothesized, for example, that the El Paso region of Texas has such a high vaccination rate due to the perceived increased risk of HPV-related cancers in the community, leading to greater voluntary vaccination uptake (22). There also may be more robust provider recommendations and vaccine outreach in areas perceived as at higher risk of HPV-related cancers. Our study affirms this reverse disparity among much of Texas but suggests that some underrepresented minority populations, notably communities of Asian residents in urban locations, may benefit from further analysis.

Comparing our results with studies from other states, we observe both similarities and differences. In New York, higher HPV vaccination rates have been reported among Hispanic and Black adolescents, aligning with our findings in Texas (23). However, these states did not exhibit the same extent of reverse disparity for male adolescents, suggesting that local cultural, socioeconomic, and policy factors might influence these patterns (23, 24). For instance, the study “Improving HPV Vaccination Rates in a Racially and Ethnically Diverse Pediatric Population” highlighted successful interventions that increased vaccination rates in a diverse population yet did not report significant gender disparities like those found in Texas (24). These differences underscore the importance of considering local context and tailored interventions when addressing HPV vaccination disparities across different regions.

Several studies have observed a reverse disparity in HPV vaccination rates among Hispanic communities, similar to trends seen in cervical cancer screening. For instance, research has shown that Hispanic adolescents have higher HPV vaccination rates compared to their non-Hispanic white counterparts, likely due to targeted public health initiatives and community outreach programs (25). Similarly, in many states, Hispanic women constitute a significant proportion of the clients served by the National Breast and Cervical Cancer Early Detection Program (NBCCEDP), indicating effective outreach and utilization of services within this community (24). Studies have also reported higher cervical cancer screening rates among Hispanic women, attributed to culturally tailored interventions and community health programs that address language barriers and provide patient navigation (26). These findings underscore the importance of culturally sensitive healthcare interventions in improving preventive health measures in Hispanic communities. However, addressing disparities in follow-up care and treatment remains critical to ensure comprehensive care for these populations.

Regarding characteristics of persistent hot spot ZIP codes of HPV vaccination coverage, such as higher rates of children on Medicaid insurance and lower rates on private insurance plans, this may be representative of the general socioeconomic makeup of the communities (i.e., lower median household income). However, this may also be due to vaccination benefits among federally sponsored insurance, notably the Vaccines for Children program, which provides the HPV vaccine at no cost to those who meet eligibility criteria (Medicaid or uninsured). The reflection on how private insurance may deter vaccination warrants a deeper discussion. Private insurance plans often have higher co-pays and deductibles compared to public insurance, which can discourage families from completing the HPV vaccination series. Studies have shown that individuals with public insurance or who are uninsured are more likely to receive vaccinations through public health programs, which often cover the full cost of vaccines (27). This financial barrier associated with private insurance plans may contribute to lower vaccination rates among insured individuals, highlighting the need for policy interventions to reduce out-of-pocket vaccination costs. More research is needed to understand the barriers to HPV vaccination among all populations, but coverage for all people, regardless of insurance status, should be prioritized.

Importantly, our study highlights differences in adolescent HPV vaccination coverage hot spots among male adolescents versus female adolescents. There were many more hot and cold spots of HPV vaccination coverage among male adolescents, indicating more clusters of areas with high HPV vaccination coverage rates and low vaccination coverage rates. This suggests that across the state, HPV vaccinations are more widespread among female adolescents compared to male adolescents. Increasing vaccination strategies specifically targeting male adolescents, therefore, may be warranted.

Our study does have some limitations. Primarily, our data is from the ImmTrac2 system, an opt-in program that may not entirely represent the population. Additionally, the use of the ACS and COI data as population-based metrics may not be representative of the individuals receiving the vaccines. We were unable to obtain more specific demographic information on participants, which limits the analysis to an ecological approach (8). Finally, there is a notable discrepancy between our reported adolescent HPV vaccination coverage rates (21–23%) and those reported in the Nehme article of (30–40%) (8), which cited higher rates for Texas. This is because the Nehme article used data from the National Immunization Survey-Teen (NIS-Teen). NIS-Teen is a random digit dialing telephone survey of households in the U.S. plus provider-reported vaccination histories of teens whose parents participate in the phone survey and consent to having their teen’s vaccination providers contacted. While NIS-Teen data offers comprehensive and representative vaccination coverage estimates, it relies on sample-based estimates. Conversely, ImmTrac2 provides detailed and timely immunization records, beneficial for monitoring and program evaluation, but its opt-in nature leads to underrepresentation and variable participation, as it requires parental consent for minors, resulting in incomplete data. Nonetheless, ImmTrac2 maintains robust coverage of vaccines given to pediatric populations (14).

To our knowledge, this is the first spatiotemporal analysis of HPV vaccination coverage in Texas. Modeling statistical hot spots of HPV vaccination and identifying ZIP codes that model as hot/cold spots 5 years in a row presents a thorough approach to understating HPV vaccination geographically in Texas. Few studies on HPV vaccinations utilize high spatial resolution data, like ZIP codes, which allow for integrating neighborhood-level data like the Child Opportunity Index and key American Community Survey variables. These data sources allow a better understanding of neighborhood context, which may affect vaccination behaviors. Understanding the spatiotemporal dynamics of HPV vaccination rates in a vast state like Texas can enable advocates to tailor messaging and outreach more effectively to promote vaccine uptake.

## Data availability statement

Publicly available datasets were analyzed in this study. This data can be found at: https://www.dshs.texas.gov/immunizations/what-we-do/programs; https://data.diversitydatakids.org/dataset/coi30-2010-tracts-child-opportunity-index-3-0-database--2010-census-tracts?_ga=2.105088003.1687438952.1713278005-6079392.1713278005.

## Author contributions

RR: Conceptualization, Data curation, Formal analysis, Funding acquisition, Methodology, Resources, Software, Supervision, Visualization, Writing – original draft, Writing – review & editing. AZ: Conceptualization, Data curation, Formal analysis, Investigation, Project administration, Resources, Supervision, Writing – original draft, Writing – review & editing. SU: Project administration, Writing – original draft, Writing – review & editing. MD: Data curation, Investigation, Methodology, Writing – review & editing. CB: Formal analysis, Funding acquisition, Methodology, Resources, Validation, Writing – review & editing.
